# Supplementary material for: Combining AI Structure Prediction and Integrative Modeling for Nanobody-Antigen Complexes
Source: J Chem Inf Model. 2026 Aug 19;66(17):11445–58. doi: 10.1021/acs.jcim.6c01301 (PMC13580113; doi:10.1021/acs.jcim.6c01301)
Supplement: Supplementary file 1 [file ci6c01301_si_001.pdf]

Supplementary Material

Combining AI structure prediction and integrative modelling for nanobody-antigen complexes

Miguel Sánchez-Marín, Marco Giulini, Alexandre M.J.J. Bonvin

Bijvoet Centre for Biomolecular Research, Faculty of Science - Chemistry, Utrecht University, Padualaan 8, 3584 Utrecht, CH, The Netherlands

Table S1 2
Table S2 3
Table S3 5
Figure S1 7
Figure S2 8
Figure S3 9
Figure S4 10
Figure S5 11
Figure S6 12
Figure S7 13
Figure S8 14
Figure S9 15
Figure S10 16
Figure S11 17
Supplementary Methods: Statistics and reproducibility 18
Supplementary References 18

Table S1. Benchmark dataset PDB IDs, CDR sequences and heavy chain species.

PDB	Nb Chain	CDR1	CDR2	CDR3	Heavy Chain Species
7tgf	B	GFTLDDY	SSSDGR	EEVCTLGIFGHGPDDY	*Vicugna pacos*
8emz	B	GRFFSSY	SWSGGS	AREGAYYPDSYYRTVRYDY	*Vicugna pacos*
8h3x	A	GFTFSRY	NSDGGR	PKRTYVPPSQFDD	*Vicugna pacos*
8gz5	B	GRTSSVY	TGNGGT	GGWGKERNYAY	*Vicugna pacos*
8cwu	B	GRTFSTS	DWSNTN	GGWGLTQPISVDY	*Lama glama*
7x2m	B	GDTLDLY	SPSGSR	SRPSAHYCSHYPTEYDD	*Vicugna pacos*
7voa	A	GFTLDYY	SGSGGI	VSHTVVAGCAFEAWTDFGS	*Vicugna pacos*
7ubx	C	GFTFSDY	NTDGS	GRVISASAIRGAV	*Camelidae*
7th2	C	PSLDYY	TGSEGS	ADPLPLVCTWGDEYDY	*Vicugna pacos*
8en2	D	GLTFSTN	NWNGGN	KMGRRLAVSRTLEEYDF	*Vicugna pacos*
8ew6	V	GFTFDDY	RIFDRH	GSFWACTRPEGAMDY	*Lama glama*
7yz9	B	GIILSIN	TAGGS	PAGRVGGT	*Vicugna pacos*
7w1s	B	ISFSSF	GIGG	WRMNDY	*Vicugna pacos*
7zmv	G	GSIFSIN	TSGGS	YGTYTLAPTGEGEYDDY	*Lama glama*
7unz	A	GSIFSTN	TSGSS	AGATIDLADFGS	*Vicugna pacos*
8en3	C	GRTDSES	NWRYAT	RYIYGSLSDSGSYDN	*Vicugna pacos*
7tgi	B	GLTLDYY	SSSDGR	DRDRLPSAITYEYNY	*Vicugna pacos*
7nxx	B	GGDTRPYITY	YTGGSG	GNGALPPGRRLSPQNMDT	*Camelus dromedarius*
8dtn	E	GFIFDSY	TSSGSS	LDYVIDGY	*Lama glama*
8h3y	E	GQTFSAW	NWNGER	MMGTYYSGSPKN	*Vicugna pacos*
7q3q	B	GLTFSSV	RWKFGN	ARVGEIIAVLISPSNYAY	*Vicugna pacos*
7tpr	E	GYTYSIC	NADGSN	HGTYDKYAPCGGFAGTYTY	*Camelus dromedarius*
7x2j	B	GRTFSRY	EWDGGT	GGNQYYSATYSIWNEYDF	*Vicugna pacos*
7b5g	D	GSIFSSN	SSRGDN	GSFYRGNYYGGSS	*Lama glama*
7x2l	B	GSISTLN	TLDGS	ENGGFFYY	*Vicugna pacos*
8dtu	A	GSTFSGY	TSSGAS	LDEGYLDYDS	*Lama glama*
8dqu	B	GLAFSMY	ISSGDS	PKFRYYFSTSPGDFDS	*Lama glama*
7uia	B	GFTFDDY	DSWSIN	EDRLGVPTINAHPSKYDYNY	*Vicugna pacos*
7t5f	F	GSIDSLY	QDGGS	KSTISTPLS	*Camelidae*
7wki	B	GLTVDDY	SSSNGS	AVSPNLECGTGPFGIYASYYGMDY	*Vicugna pacos*
7x7e	A	GGTLASF	DVINR	HFVPPGSRLRGCLVNELYNY	*Lama*
7qbg	E	GFTPGIY	SSRGSS	IYQPSNGCVLRPEYSY	*Vicugna pacos*
7wd2	D	GFTLDYY	SSNNS	EPDYSGVYYYTCGWTDFGS	*Vicugna pacos*
7qne	G	GRTFTAY	DQGRI	GAGFWGLRTASSYHY	*Lama glama*
7r24	E	GRTFSAY	SWSGNS	RKPMYRVDISKGQNYDY	*Vicugna pacos*
7r24	C	GRTSGAL	WWNDGT	RTPSSQTLY	*Vicugna pacos*
7xqv	B	GDTWWSS	SFYPTEY	IAWGPWMRTSWY	*Camelus bactrianus*
7whi	G	DFYFDYY	SGLGGA	RSPFGDYAFSY	*Homo sapiens*
8bb7	D	GFRLDYY	SSSGGS	SSYNTQRAECYGMDY	*Camelidae*
8en0	D	GRTFSSY	TGSGDS	YRTGGPPQ	*Vicugna pacos*

Table S2. Benchmark dataset antigen species and PDB from the used homologous antigens for docking. AF Multimer indicates the antigens that were modelled using AlphaFold2-Multimer.

PDB	Ag Chain	Antigen Species	Antigen Name	PDB (chain) used for docking
**7tgf**	A	*Ricinus communis*	Ricin Chain A	7y07 (A)
**8emz**	A	*Norovirus*	Gii.17 P Domain	5lkk (A)
**8h3x**	C	*Bacteroides fragilis*	Fragilysin	AF Multimer
**8gz5**	A	Severe Acute Respiratory Syndrome Coronavirus2	Spike Protein S1	7sxt (A)
**8cwu**	A	Severe Acute Respiratory Syndrome Coronavirus2	Spike Protein S1	7z8o (A)
**7x2m**	E	Severe Acute Respiratory Syndrome Coronavirus2	Spike Protein S1	AF Multimer
**7voa**	B	Severe Acute Respiratory Syndrome Coronavirus2	Spike Glycoprotein	7eam (A)
**7ubx**	B	*Clostridioides difficile*	Toxin A	7u1z (A)
**7th2**	A	*Ricinus communis*	Ricin A Chain	AF Multimer
**8en2**	A	*Norovirus*	Gii.10 P Domain	AF Multimer
**8ew6**	W	*Homo sapiens*	T-Cell Surface Glycoprotein Cd8 Alpha Chain	AF Multimer
**7yz9**	A	*Mycobacterium tuberculosis H37rv*	Adenylate Cyclase	AF Multimer
**7w1s**	A	Severe Acute Respiratory Syndrome Coronavirus2	Spike Protein S1	7eam (A)
**7zmv**	A	*Homo sapiens*	Atp-Dependent Dna Helicase Q5	AF Multimer
**7unz**	D	*Plasmodium falciparum*	Cysteine-Rich Small Secreted Protein Css, Putative	7uny (A)
**8en3**	A	*Norovirus*	Capsid Protein Vp1	8emz (A)
**7tgi**	A	*Ricinus communis*	Ricin Chain A	7y07 (A)
**7nxx**	A	*Homo sapiens*	Superoxide Dismutase [Cu-Zn]	2c9v (A)
**8dtn**	F	*Homo sapiens*	B-Cell Lymphoma/Leukemia 11a	AF Multimer
**8h3y**	C	*Bacteroides fragilis*	Fragilysin	AF Multimer
**7q3q**	A	Severe Acute Respiratory Syndrome Coronavirus2	Spike Glycoprotein	7q3r (A)
**7tpr**	C	Severe Acute Respiratory Syndrome Coronavirus2	Spike Glycoprotein	7z8o (A)
**7x2j**	S	Severe Acute Respiratory Syndrome-Related Coronavirus	Spike Protein S1	7zh1 (A)
**7b5g**	C	*Escherichia coli K-12*	Lexa Repressor	7ozj (A)
**7x2l**	E	Severe Acute Respiratory Syndrome Coronavirus2	Spike Protein S1	7z8o (A)
**8dtu**	C	*Homo sapiens*	B-Cell Lymphoma/Leukemia 11a	8dtn (F)
**8dqu**	F	Severe Acute Respiratory Syndrome Coronavirus2	Non-Structural Protein 9	AF Multimer
**7uia**	A	*Clostridium botulinum*	Neurotoxin Type E	7ovw (A)
**7t5f**	D	*Clostridium botulinum*	Botulinum Neurotoxin Type B	7na9 (A)
**7wki**	A	*Homo sapiens*	Complement Factor H	3sw0 (X)

**7x7e**	D	Severe Acute Respiratory Syndrome Coronavirus2	Spike Protein S1	7z8o (A)
**7qbg**	C | B	*Homo sapiens* | *Homo sapiens*	Transcobalamin-2 | Cd320 Antigen	AF Multimer
**7wd2**	B	Severe Acute Respiratory Syndrome Coronavirus2	Spike Protein S1	AF Multimer
**7qne**	A | E	*Homo sapiens* | *Homo sapiens*	Gaba(A) Receptor Subunit Alpha-1 | Gamma-Aminobutyric Acid Receptor Subunit Beta-3	AF Multimer (considered failed)
**7r24**	A	*Rattus norvegicus*	Activity-Regulated Cytoskeleton-Associated Protein	AF Multimer
**7xqv**	A	*Rattus norvegicus*	Rhoa	AF Multimer
**7whi**	A	Severe Acute Respiratory Syndrome Coronavirus2	Spike Glycoprotein	7x66 (R)
**8bb7**	B	*Mus musculus*	Plexin-B1	AF Multimer
**8en0**	A	*Norovirus*	Capsid Protein Vp1	8emz (A)

Table S3. Alphafold modelling and docking results summary for the benchmark dataset. Alphafold and HADDOCK results are reported over the top 10 structures. HADDOCK results come from IBMu ensemble with unbound antigen, true interface scenario and Flexible Refinement stage. IBMu ensemble size is reported as the number of models between brackets in the Max HADDOCK DockQ column.

PDB	Nb Angle Class	Min CDR3 RMSD (Å)	Unbound Epitope RMSD (Å)	Max AF2 DockQ (max ipTM)	Max AF3 DockQ (max ipTM)	Max HADDOCK DockQ (ensemble size)	HADDOCK Capri class
7tgfB	Extended	1.738	0.463	0.863 (0.88)	0.725 (0.90)	0.819 (5)	Medium
8emzB	Kinked	2.192	1.256	0.385 (0.15)	0.380 (0.11)	0.669 (6)	Medium
8h3xA	Other	2.420	0.611	0.448 (0.44)	0.796 (0.86)	0.604 (7)	Medium
8gz5B	Kinked	1.020	0.441	0.153 (0.27)	0.895 (0.85)	0.814 (2)	Medium
8cwuB	Kinked	2.541	0.791	0.050 (0.20)	0.757 (0.80)	0.294 (5)	Acceptable
7x2mB	Kinked	1.470	1.124	0.032 (0.36)	0.038 (0.18)	0.245 (2)	Acceptable
7voaA	Kinked	1.740	0.557	0.093 (0.38)	0.051 (0.14)	0.400 (7)	Acceptable
7ubxC	Extended	0.989	4.607	0.032 (0.20)	0.920 (0.91)	0.516 (11)	Acceptable
7th2C	Kinked	2.693	0.393	0.200 (0.54)	0.540 (0.16)	0.362 (3)	Acceptable
8en2D	Kinked	1.171	0.398	0.815 (0.75)	0.899 (0.82)	0.587 (5)	Acceptable
8ew6V	Kinked	1.414	2.561	0.045 (0.73)	0.131 (0.19)	0.546 (6)	Medium
7yz9B	Other	3.285	1.191	0.113 (0.16)	0.857 (0.86)	0.182 (5)	Incorrect
7w1sB	Failed	1.365	0.681	0.789 (0.40)	0.619 (0.25)	0.723 (2)	Medium
7zmvG	Extended	1.066	1.680	0.035 (0.23)	0.888 (0.70)	0.566 (6)	Medium
7unzA	Other	4.543	1.036	0.210 (0.17)	0.505 (0.23)	0.127 (11)	Incorrect
8en3C	Kinked	2.684	1.753	0.138 (0.17)	0.221 (0.11)	0.311 (3)	Acceptable
7tgiB	Kinked	1.157	0.585	0.863 (0.92)	0.850 (0.92)	0.854 (7)	High
7nxxB	Kinked	2.562	0.565	0.169 (0.19)	0.142 (0.18)	0.391 (4)	Acceptable
8dtnE	Other	5.189	3.534	0.311 (0.34)	0.162 (0.39)	0.236 (4)	Acceptable
8h3yE	Kinked	0.565	3.575	0.023 (0.60)	0.036 (0.15)	0.635 (3)	Medium
7q3qB	Kinked	0.529	0.571	0.915 (0.85)	0.948 (0.89)	0.870 (3)	High
7tprE	Extended	2.374	2.192	0.070 (0.25)	0.153 (0.36)	0.331 (7)	Medium
7x2jB	Kinked	2.410	1.223	0.025 (0.26)	0.924 (0.90)	0.436 (8)	Acceptable
7b5gD	Kinked	1.240	0.964	0.042 (0.21)	0.114 (0.16)	0.766 (8)	Medium
7x2lB	Extended	0.784	0.779	0.849 (0.86)	0.834 (0.79)	0.721 (3)	Medium
8dtuA	Kinked	4.090	5.644	0.174 (0.50)	0.130 (0.60)	0.317 (6)	Acceptable
8dquB	Kinked	0.597	5.690	0.404 (0.78)	0.399 (0.57)	0.375 (3)	Incorrect
7uiaB	Kinked	3.485	2.220	0.041 (0.21)	0.671 (0.60)	0.079 (8)	Incorrect
7t5fF	Extended	1.778	0.570	0.021 (0.26)	0.154 (0.11)	0.641 (2)	Medium
7wkiB	Kinked	3.221	3.716	0.194 (0.19)	0.144 (0.13)	0.140 (7)	Incorrect
7x7eA	Kinked	1.283	0.875	0.035 (0.66)	0.029 (0.18)	0.586 (5)	Medium
7qbgE	Kinked	2.326	2.520	0.224 (0.62)	0.206 (0.57)	0.030 (2)	Incorrect
7wd2D	Kinked	1.617	0.579	0.029 (0.25)	0.911 (0.85)	0.283 (6)	Acceptable
7qneG	Kinked	2.106	13.376	0.025 (0.62)	0.852 (0.80)	0.130 (1)	Incorrect

**7r24E**	Kinked	1.113	1.589	0.752 (0.86)	0.784 (0.87)	0.478 (2)	Medium
**7r24C**	Extended	2.001	1.375	0.027 (0.65)	0.115 (0.25)	0.243 (3)	Incorrect
**7xqvB**	Extended	4.077	0.632	0.176 (0.32)	0.149 (0.64)	0.641 (7)	Medium
**7whiG**	Kinked	0.946	1.639	0.006 (0.29)	0.005 (0.18)	0.529 (2)	Medium
**8bb7D**	Kinked	1.387	1.206	0.018 (0.18)	0.019 (0.13)	0.399 (4)	Acceptable
**8en0D**	Extended	3.555	2.169	0.035 (0.15)	0.028 (0.14)	0.353 (4)	Acceptable

Figure S1. Benchmark dataset variability analysis. a) Nanobody CDR3 length distribution in the benchmark dataset. b) Antigen size distribution in the benchmark dataset. c) Pairwise epitope structural comparison by TM-score in the benchmark dataset. Axes show nanobody PDB IDs. Red square shows the minimum value (0.018) and green square shows the maximum value (0.717).

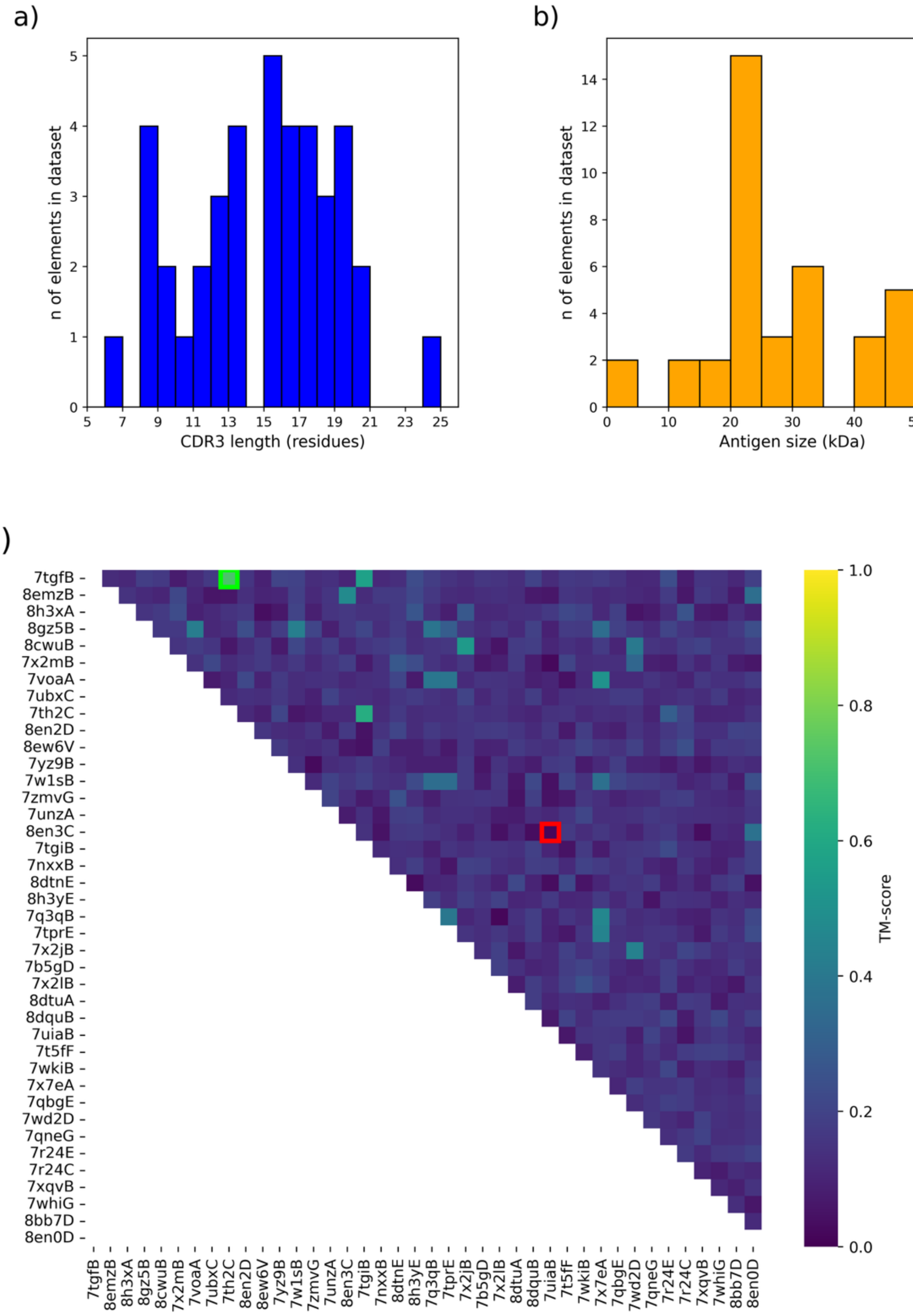

Figure S2. Violin plot showing the distribution of the backbone CDR3 RMSD from the best ranked nanobody prediction for the different methods. The prediction methods shown in the plot are AlphaFold2-Multimer (AF Multimer), AlphaFold2 (AF Monomer), ImmuneBuilder (IB), RaptorX-Single (RXS), NanoNet (NN) and AlphaFold3 (AF3). Median values are indicated in the plot.

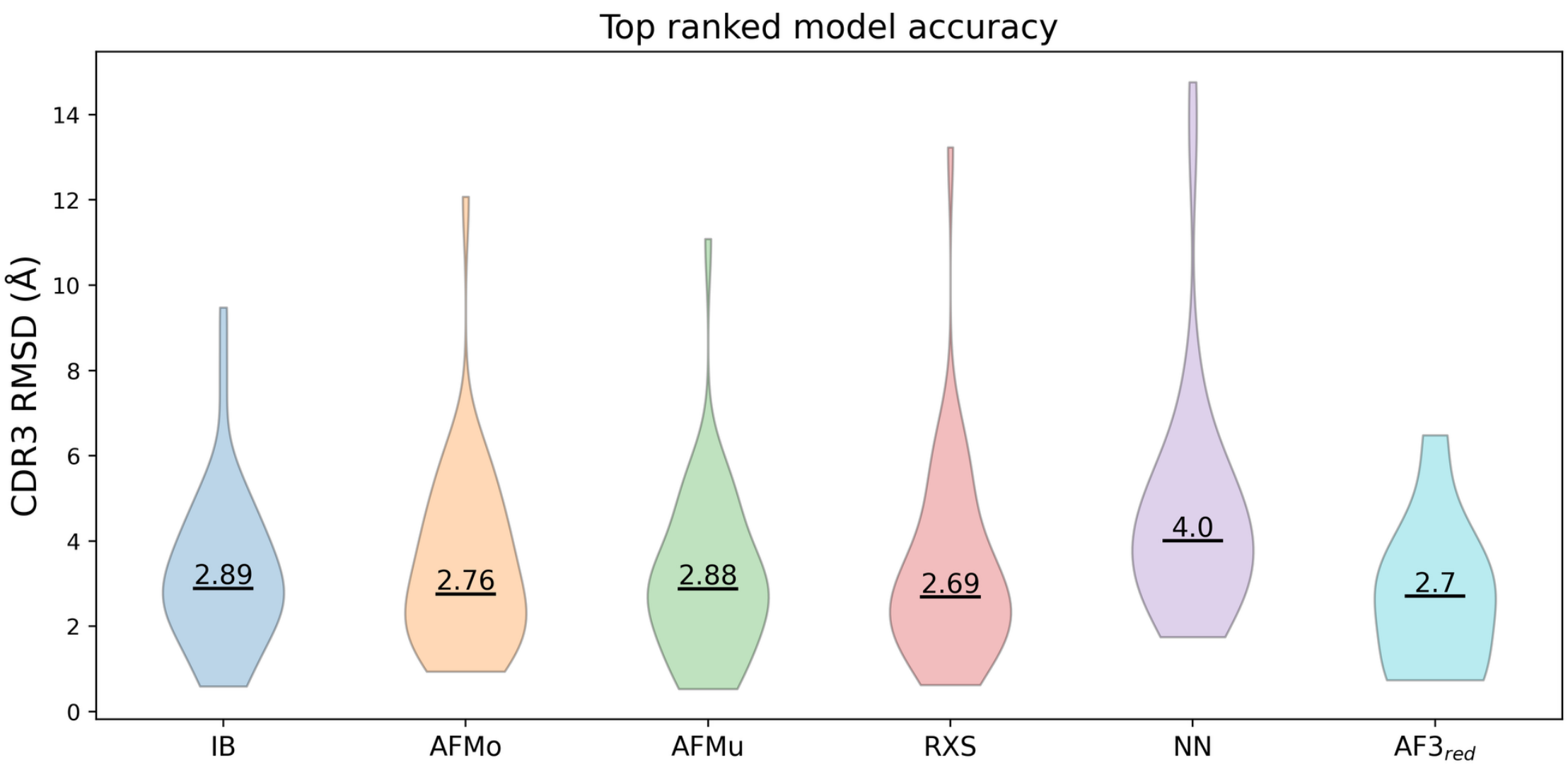

Figure S3. Bar plots showing docking success rate (SR) for All Surface scenario as a function of the TopN ranked structures. The results shown are coming from the input ensemble of ImmuneBuilder + AlphaFold2-Multimer (IBMu) nanobody models and the bound antigen structures. The left plot shows results after Rigid Body stage of the pipeline, center left plot after Flexible Refinement (Flexref) stage and center right plot after Energy Minimization (Emref) stage. AlphaFold2-Multimer (AF2M) and Alphafold3 (AF3) results are shown in the right panel. The color scheme indicates the quality of the represented models following CAPRI criteria. From the plot we can observe how AF2M and AF3 outperform HADDOCK when no information is available and should be always preferred in this scenario. In terms of docking, the success rate is higher at the rigid-body docking stage.

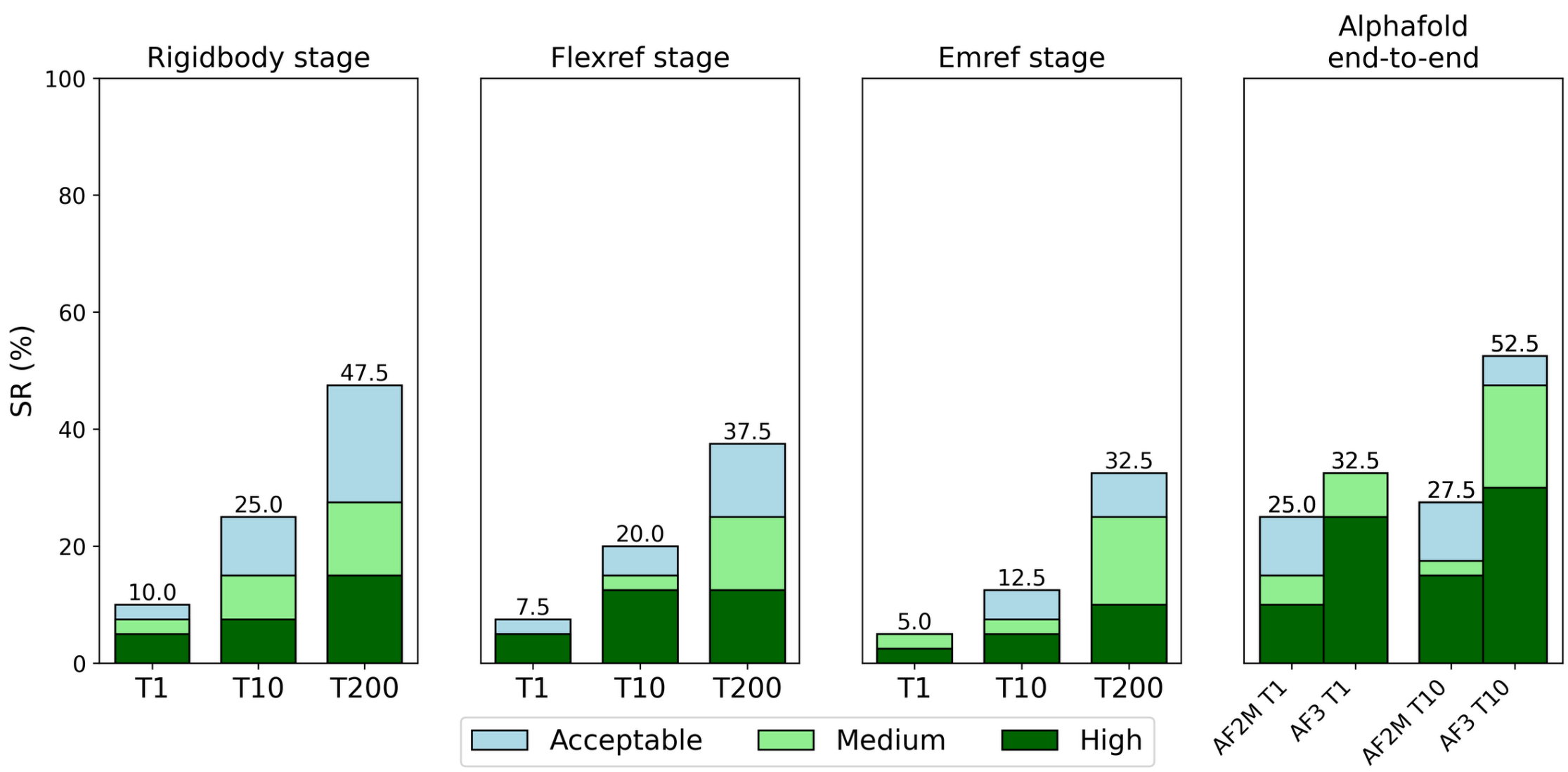

Figure S4. Plots relating DockQ in HADDOCK Top10 models with the percentage of true epitope residues defined in the two-hit interface (2I) scenario restraints. Results are shown for IBMu ensemble using both bound and unbound antigen structures. The percentage of true epitope residues present in the 2I restraints was calculated dividing the number of residues defined in the 2I restraints that are actually present in the epitope of the reference structure by the total number of residues defined in the restraints.

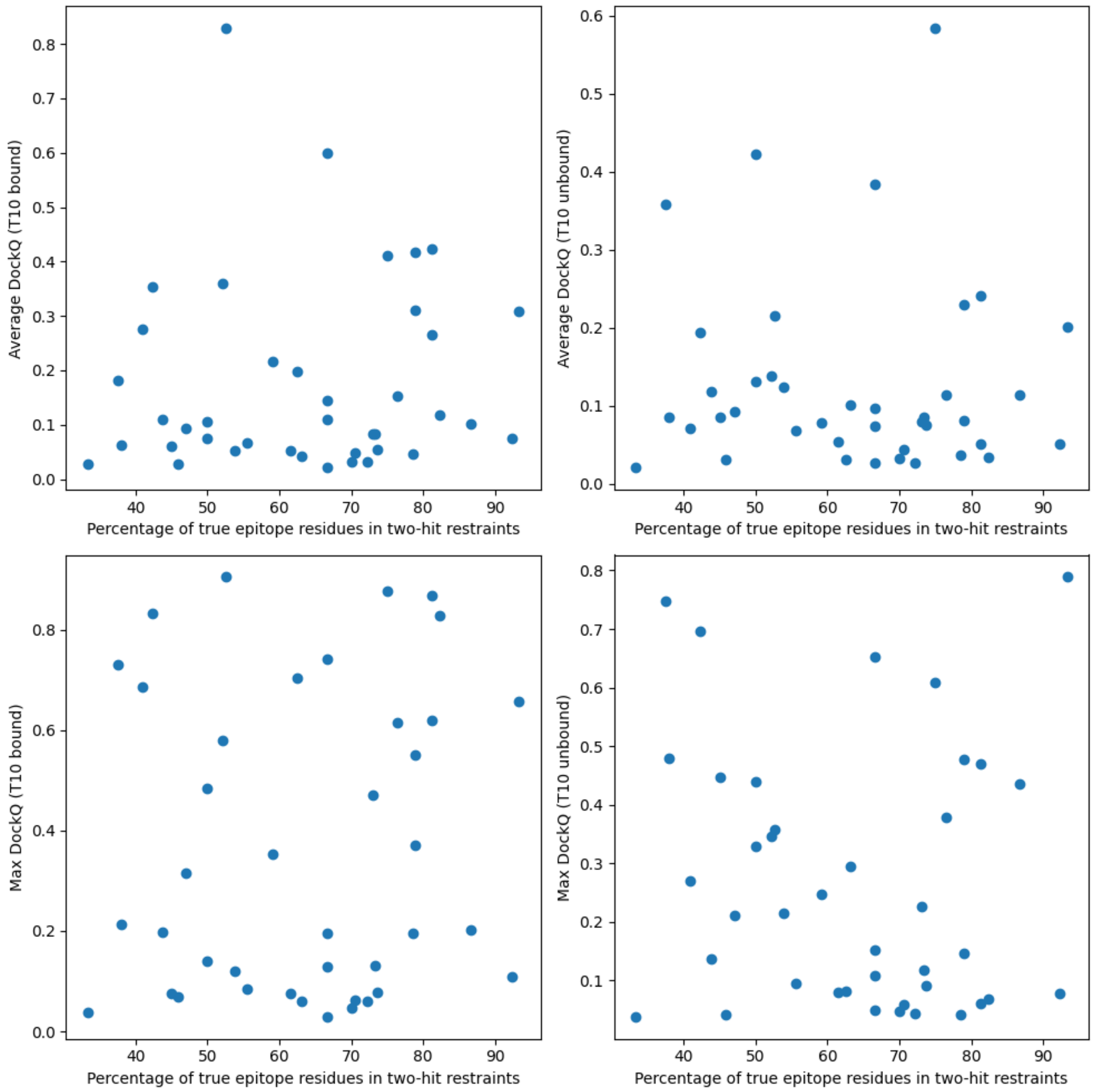

Figure S5. VoroIF-GNN scoring does not clearly improve the HADDOCK scoring of nanobody-antigen models. Bar plot comparing model docking success rates (SRs) for TopN structures after Voronoi scoring (VS) and HADDOCK scoring (HS) for different information scenarios. Data shown comes from the input ensemble of ImmuneBuilder + AlphaFold2-Multimer nanobody models and unbound antigen structures. All considered models were scored after the Energy Minimization stage. The color scheme shows the quality of the represented models following CAPRI criteria.

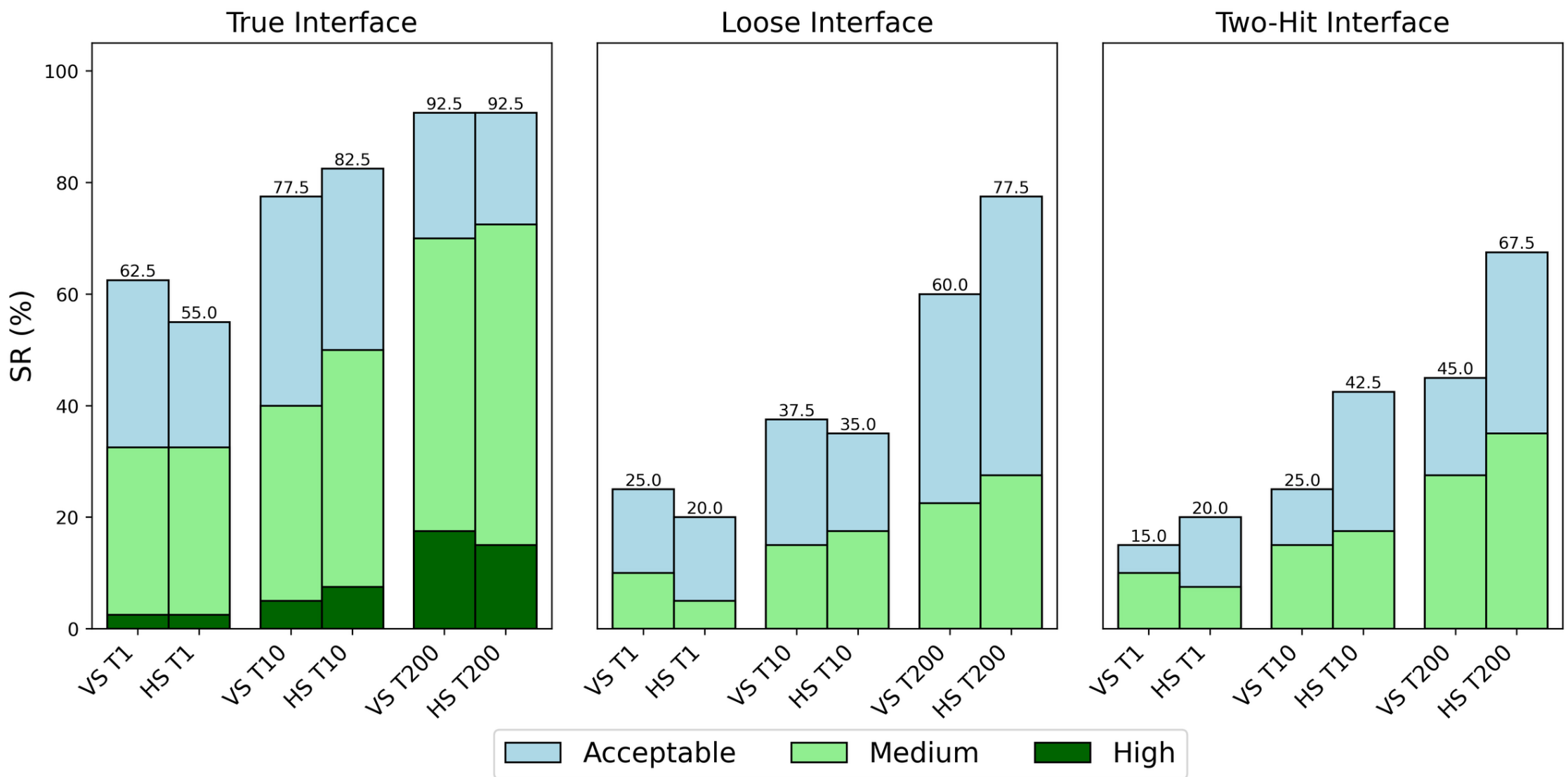

Figure S6. Scatter plot showing the relation between maximum DockQ among Top10 nanobody-antigen models and epitope RMSD. The presented data come from the True Interface scenario runs with unbound antigen structures (excluding PDB ID 7QNE, for which unbound runs were not performed). The plotted models were retrieved after Energy Minimization stage. Represented nanobody structure ensembles are ImmuneBuilder (IB), ImmuneBuilder + AlphaFold2 (IBMo), ImmuneBuilder + AlphaFold2-Multimer (IBMu) and ImmuneBuilder + AlphaFold2 + AlphaFold2-Multimer (IBMM). Pearson correlation coefficients are shown for each ensemble. The horizontal dashed line indicates a DockQ score of 0.23 which is the cutoff for an acceptable model.

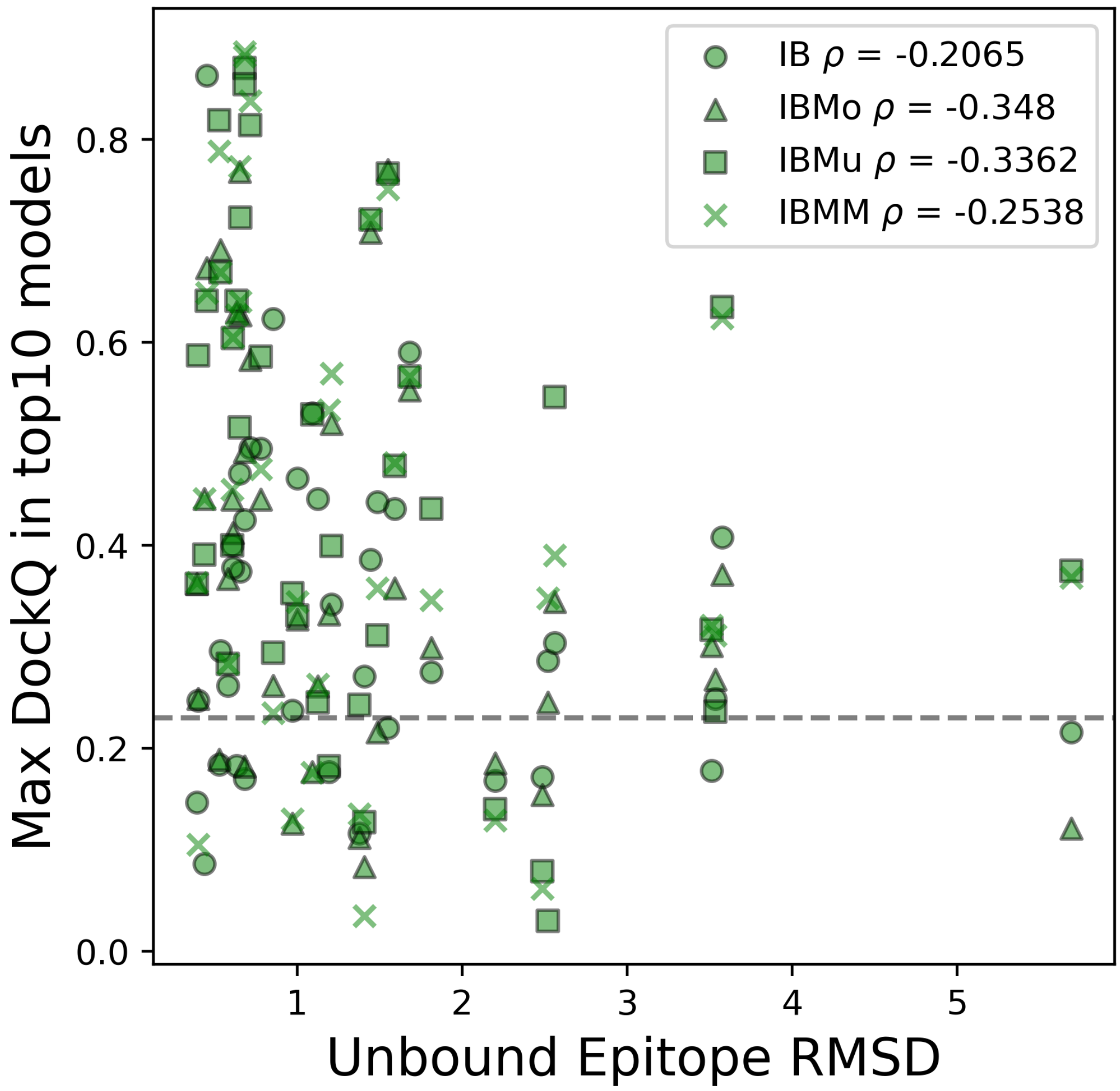

Figure S7. HADDOCK3 performance on nanobody-antigen docking for 'Kinked' and 'Extended' nanobodies. From the original benchmark dataset 29 nanobodies are classified as 'Kinked' and 9 as 'Extended'. HADDOCK success rates (SRs) for nanobody-antigen docking models after Flexible Refinement stage for True, Loose, and Two-Hit Interface scenarios. Docking success is reported for the ensembles ImmuneBuilder (IB) and ImmuneBuilder + AlphaFold2-Multimer (IBMu), both for bound (B) and unbound antigens (U) for Top 10 ranked models. For IBMu we report also the cluster-based success rate. Clusters were defined based on Fraction of Common Contact (FCC) clustering of the models at the end of the workflow. Success is considered when at least one of the four first-ranked models in any of the Top10 ranked clusters is classified as acceptable/medium/high quality. The AlphaFold2-Multimer (AF2M) and AlphaFold3 (AF3) SRs for Top10 structures are provided on the right. Light blue, light green, and dark green indicate acceptable, medium, and high-quality models, respectively, as defined by the CAPRI criteria. Acceptable SRs (%) are shown on top of the bars.

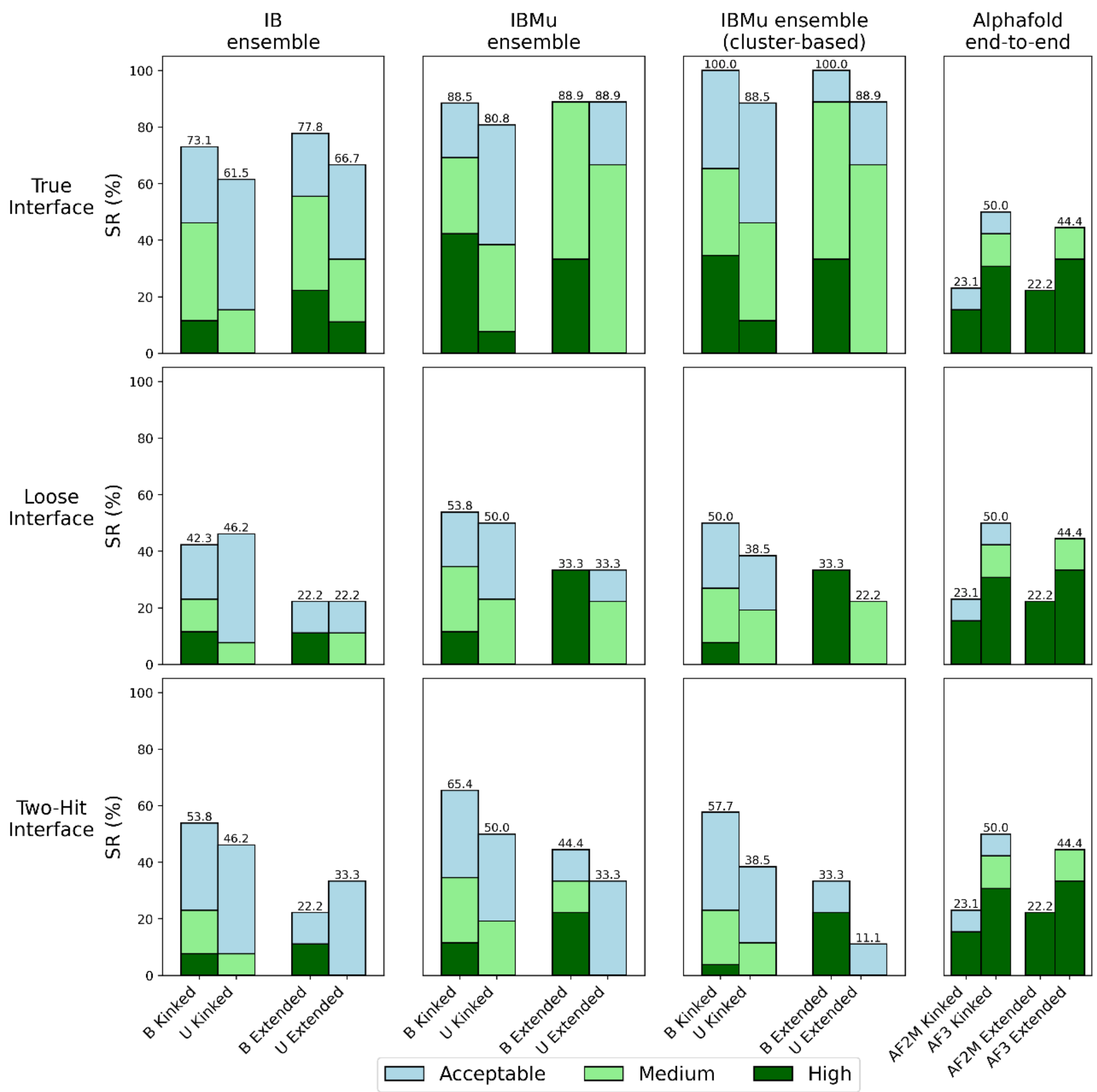

Figure S8. Percentage of 'kinked' and 'extended' CDR3 conformation structures that involve each nanobody region in antigen binding, calculated over the Paratope Dataset (see main text). Structures that did not belong to any of the two classes are not considered in this analysis. Chi-squared significance in contingency tests between both classes for each region is labelled (*p*-values FR2: 0.0015; FR3: 0.012).

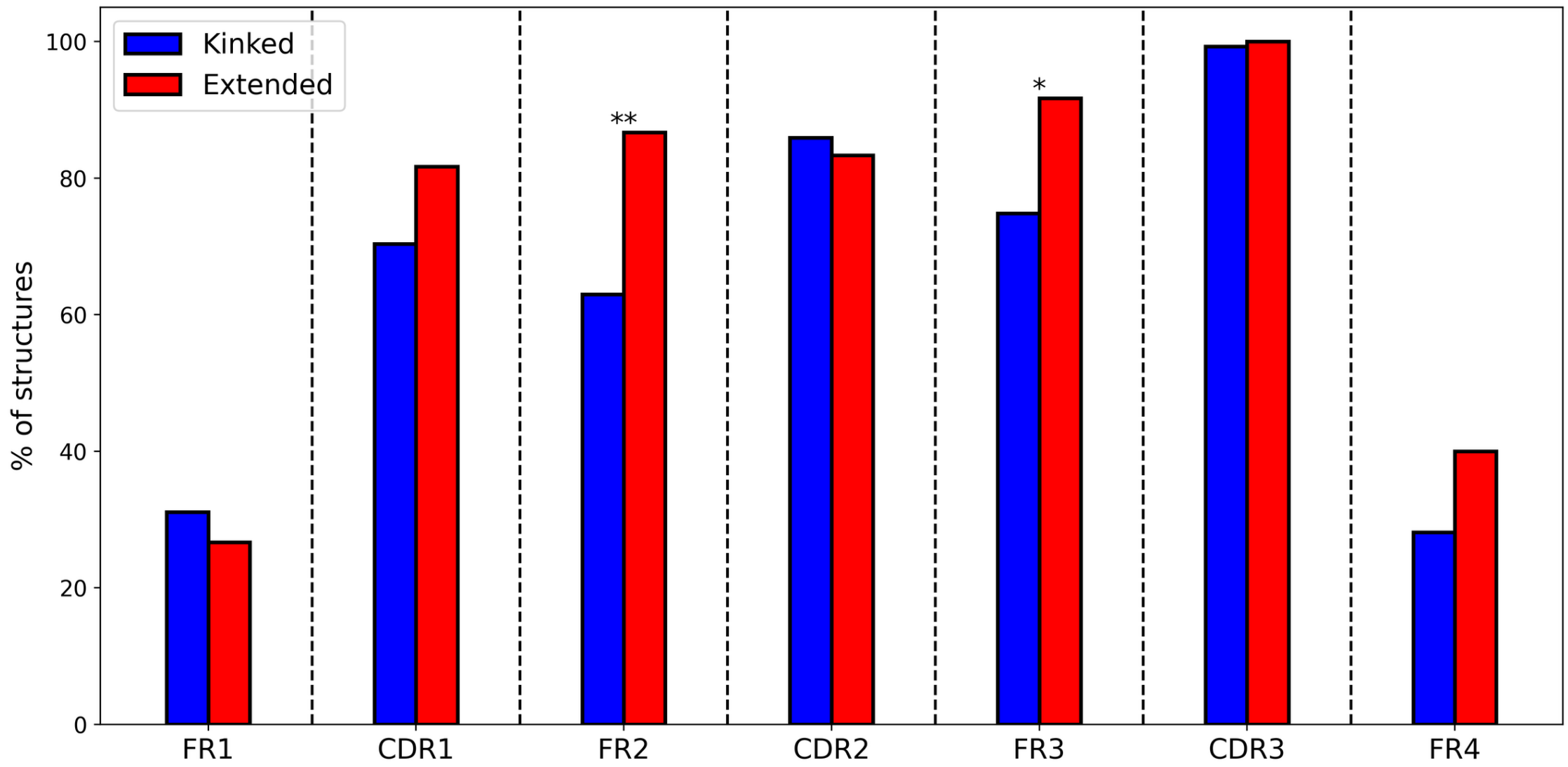

Figure S9. Bar plots showing the docking success rates for the IBMu ensemble applying the mixed paratope restraints, namely combining CDR and FR restraints. The two graphs show Loose Interface and Two-Hit Interface scenarios, using bound (B) or unbound (U) antigens. The color scheme shows the quality of the represented models following CAPRI criteria. Acceptable SR is plotted on top of the bars.

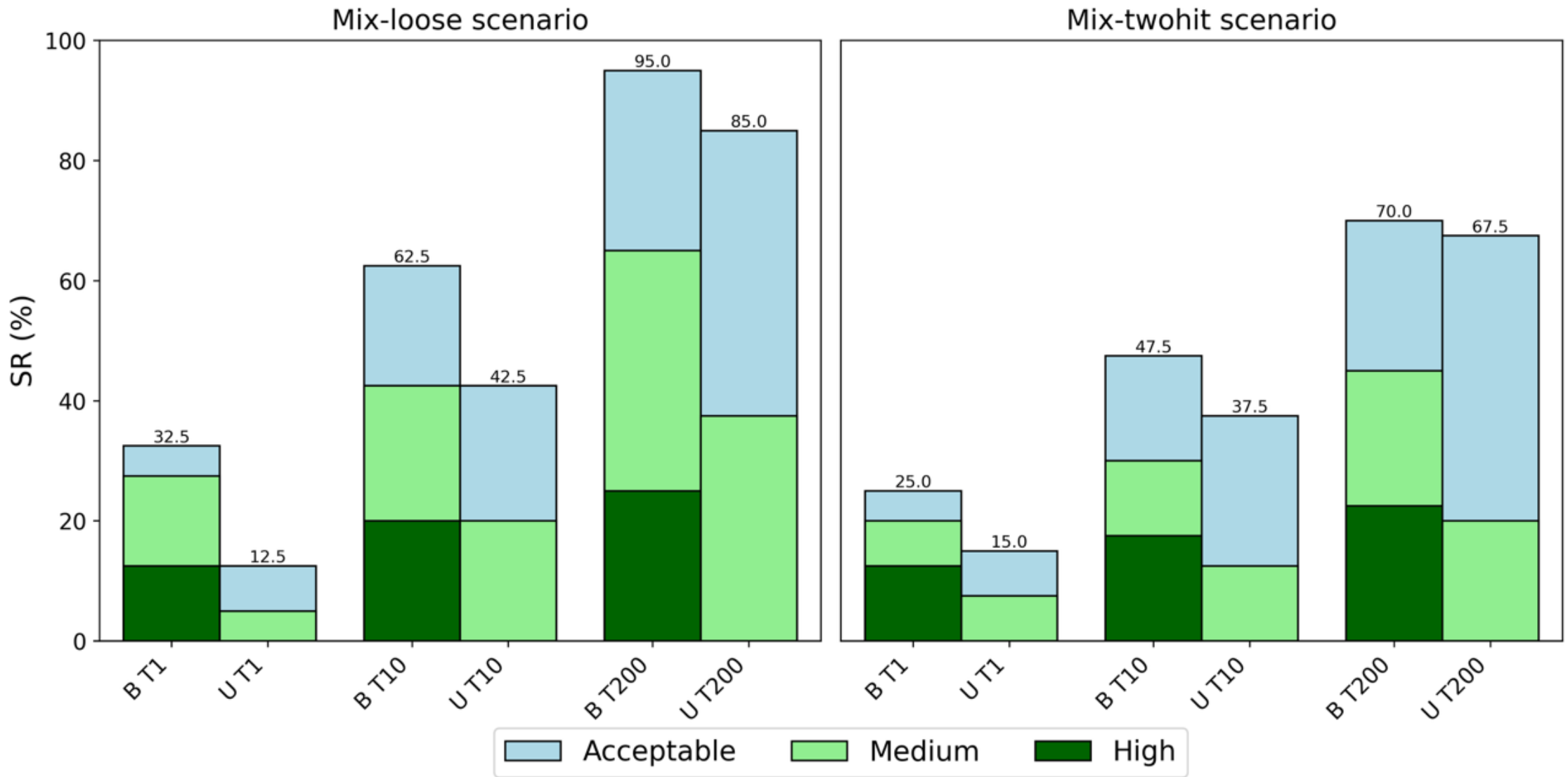

Figure S10. Example workflow for the energy minimization refinement of nanobody or antigen structures from AlphaFold methods.

```
# ===========================================================================

run_dir = “emref_7q3qB_af_multimer_centroids"
molecules = "7q3qB_af_multimer_centroids_ensemble.pdb”
clean = false
# ===========================================================================

[topoaa]

[emref]
```

Figure S11. Example workflow of the default nanobody-antigen HADDOCK pipeline.

```
# ================================================================

run_dir = "nanobody_antigen_docking"
mode = “local”

molecules = ["7q3qB_ib_af_multimer_ensemble.pdb”,
             “7q3q_B_A_alphafold2_multimer_antigen.pdb"]
# ================================================================
[topoaa]
[rigidbody]
tolerance = 5
ambig_fname = "7q3qB_real_ambig.tbl"
[caprieval]
reference_fname = " 7q3q-B:A_ref.pdb"
[seletop]
select = 200
[caprieval]
reference_fname = " 7q3q-B:A_ref.pdb"
[flexref]
tolerance = 5
ambig_fname = "7q3qB_real_ambig.tbl"
[caprieval]
reference_fname = " 7q3q-B:A_ref.pdb"
[emref]
tolerance = 5
ambig_fname = "7q3qB_real_ambig.tbl"
[caprieval]
reference_fname = " 7q3q-B:A_ref.pdb"
[clustfcc]
[seletopclusts]
top_models = 4
[caprieval]
reference_fname = " 7q3q-B:A_ref.pdb"
# ================================================================
```

Supplementary Methods: Statistics and reproducibility

We calculated statistical differences using Chi-squared test using adjusted p-values. Significance levels were labelled as: n.s (non-significant), *(p <=0.05), **(p <=0.01), ***(p<=0.001). Statistical tests were performed using scipy Python package [1].

Supplementary References

1. Virtanen, P. *et al.* SciPy 1.0: fundamental algorithms for scientific computing in Python. *Nat Methods* **17**, 261–272 (2020).
